# Flavonoid-Mediated Suppression of Tumor Angiogenesis: Roles of Ang-Tie/PI3K/AKT

**DOI:** 10.3390/pathophysiology31040043

**Published:** 2024-10-12

**Authors:** Shallu Saini, Hardeep Singh Tuli, Reena V. Saini, Adesh K. Saini, Katrin Sak, Damandeep Kaur, Moyad Shahwan, Ritu Chauhan, Abhishek Chauhan

**Affiliations:** 1 Department of Bio-Sciences and Technology, Maharishi Markandeshwar Engineering College, Maharishi Markandeshwar (Deemed to Be University), Mullana, Ambala 133207, India; reenavohra10@mmumullana.org (R.V.S.); sainiade@gmail.com (A.K.S.); 2 NGO Praeventio, 50407 Tartu, Estonia; katrin.sak.001@mail.ee; 3 University Centre for Research and Development, Chandigarh University, Mohali 140413, India; deepdaman602@yahoo.com; 4 Department of Clinical Sciences, College of Pharmacy and Health Sciences, Ajman University, Ajman 4184, United Arab Emirates; moyad76@hotmail.com; 5 Centre of Medical and Bio-Allied Health Sciences Research, Ajman University, Ajman 346, United Arab Emirates; 6 Department of Biotechnology, Graphic Era Deemed to Be University, Dehradun 248002, India; rituachauhan25@gmail.com; 7 Amity Institute of Environmental Toxicology, Safety and Management, Amity University, Sector-125, Noida 201303, India; akchauhan@amity.edu

**Keywords:** angiogenesis, anti-cancerous, flavonoid, metabolism, tumor, phytocompound

## Abstract

Angiogenesis is a process involved in the formation of new blood capillaries from pre-existing ones. It is regulated by several anti-angiogenic molecules involved in tumor growth and metastasis. The endothelial angiopoietin Ang-Tie/PI3K/AKT growth receptor pathway is necessary for healthy vascular development. The activation of AKT is controlled by a multistep process involving phosphoinositide 3-kinase (PI3K). This article aims to provide an overview of the role and mechanism of the Ang-Tie/PI3K/AKT signaling pathways and the potential of flavonoids as anti-angiogenic drugs. Flavonoids have shown great potential in preventing angiogenesis by targeting signaling pathways and exhibit additional anti-cancer properties. Research studies have revealed that the currently available anti-angiogenic drugs do not meet the safety and efficacy standards for treating tumor growth. Phytocompounds have long been a valuable resource for the development of novel therapeutic drugs. This article explores recent findings explaining the role and mechanism of the Ang-Tie/PI3K/AKT signaling pathways, as well as the interaction of flavonoids with angiogenic signaling pathways as a novel therapeutic approach. Several investigations have shown that synergistic studies of natural phytocompounds have great potential to target these pathways to inhibit tumor growth. Therefore, flavonoid-based medications may offer a more effective synergistic strategy to treat cancer.

## 1. Introduction

Angiogenesis is a process of generation of new blood vessels from the pre-existing vasculature. Blood vasculature is essential to supply nutrients and oxygen to the microenvironment of the tissue [1]. Cancer cells have low oxygen levels, i.e., hypoxia, which lowers the chance of patients’ survival. Hypoxia triggers angiogenesis, and the cells respond by releasing hypoxia-inducible factor (HIF). Therefore, it is a critical process in carcinogenesis involving many important sequential steps, such as migration, proliferation, and tube formation by endothelial cells. Many angiogenic factors work together in a highly coordinated manner to promote the formation of functional capillaries and the expansion of endothelial cells [2]. Vascular endothelial growth factor (VEGF-A) and fibroblast growth factor (FGF) are the angiogenic factors that cause these cells to respond [3]. As a result, VEGF-A and VEGFR-2 promote endothelial cell invasion, migration, and tube formation [4]. Furthermore, cancer angiogenesis is known to facilitate tumor metastasis by activating matrix metalloproteinases (MMPs). The release of MMPs causes the basement membrane to break down, allowing the cells to move in the direction of the angiogenic signals [5]. The maturation of newly formed vasculature is characterized by mural cells (pericytes and vascular smooth muscle cells), via suppression of endothelial proliferation and capillary migration. This process is regulated by factors such as angiopoietins (Ang1 and Ang2) and transforming growth factor-beta (TGF-β). The four identified angiopoietins are members of a secreted protein family that binds to the Tie receptor. Tie receptors and angiopoietins have a major impact on angiogenesis. VEGFRs and the two tyrosine kinase Tie receptors, Tie1 and Tie2, have similar expression patterns [6]. The PI3/AKT signaling pathway plays an important role in cell survival, proliferation, and angiogenesis through Ang1-mediated signaling [7]. The activation of the PI3/AKT signaling pathway has been shown to occur downstream of Tie2, which is necessary for cell survival effects [8]. 

**Figure 1:**
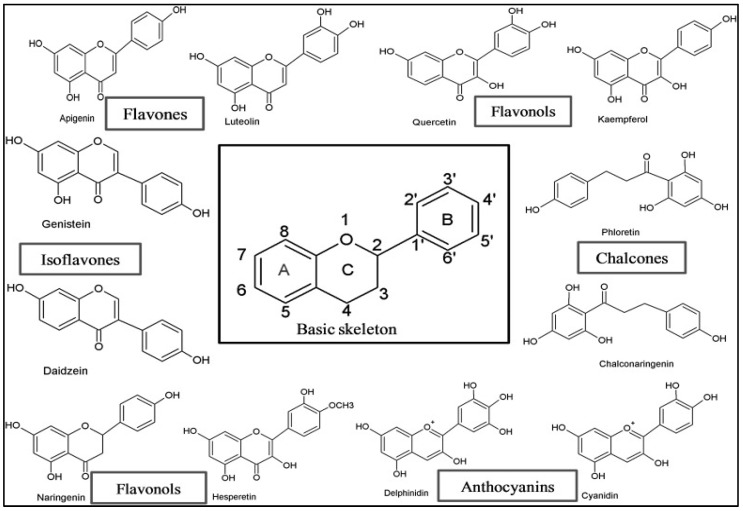
The general structure of Flavonoids and their subclasses [9].

Flavonoids are a broad class of secondary metabolites with a polyphenolic structure, commonly found in a variety of foods, fruits, vegetables, and drinks. Flavonoids consist of a fifteen-carbon skeleton with two benzene rings joined by a connecting three-carbon chain. They are therefore referred to as C6-C3-C6 phytocompounds. The chemical structure, level of oxidation, and unsaturation of the connecting chain are responsible for classifying flavonoids (C3). The different types of flavonoids include the following: anthocyanidins, flavones, flavonols, flavanones, flavan-3-ols, flavanonols, and isoflavones [Figure 1]. Dietary sources of flavonoids include buckwheat, onions, parsley, blueberries and other berries, Ginkgo biloba, bananas, all citrus fruits, black tea, green tea, oolong tea, and dark chocolate with a minimum cocoa content of 70% [9]. According to Tosetti et al. (2002), flavonoids have been identified as potent inhibitors of angiogenesis. Due to their anti-angiogenic capabilities, these inhibitors can prevent the passage of nutrients and oxygen to growing malignant cells, ultimately leading to cell death [10]. Recent research has highlighted the cooperative and complementary functions of angiopoietins with Tie2 and PI3K/AKT during angiogenesis [11]. Several research investigations have demonstrated that natural bioactive compounds, such as polyphenols, show great potential in treating various disorders [12]. Previous studies by Subbaraj et al. (2021) and Wei and Zhang (2024) reviewed the anti-angiogenic effects of flavonoids by targeting various pathways, such as NF-κB, PI3-K/Akt, ERK1/2, HIF-1α/VEGF/VEGFR2/PI3K/AKT, Wnt/β-catenin, JNK1/STAT3, and MAPK/AP-1 angiogenic signaling [13,14]. In contrast, the current study focused on the less-explored Ang-Tie/PI3/AKT signaling pathway and the molecular mechanisms underlying the anti-angiogenic activities of flavonoids.

## 2. Signaling Proteins Involved in Angiogenesis

Various proteins are involved in angiogenic induction, including the vascular endothelial growth factor (VEGF) family, angiopoietins (ANG), transforming growth factor-beta (TGF-β), platelet-derived growth factor, tumor necrosis factor-alpha (TNF-α), interleukins, and the fibroblast growth factor (FGF) family. Angiogenesis and tumor metastasis are regulated by growth factors, which initiate, control, and terminate this complex process [15].

### 2.1. Angiopoietin-1 (Ang1)

A small class of growth factor ligands for the endothelium-specific receptor tyrosine kinase known as Tie2 (tyrosine kinase with immunoglobulin-like and EGF-like domains) are referred to as angiopoietins. They are crucial for both normal and pathological angiogenesis [16]. The structure of Ang1, along with Ang2 and Ang3/4, shows that Ang1 is an oligomeric glycoprotein that is released and is a member of the angiopoietin group of growth factors. These ligands target Tie2, one of the receptor tyrosine kinases, while the other, Tie1, is predominantly expressed in the vascular endothelium [17]. Angiopoietin-1 (Ang1) acts as a Tie2 agonist, while Ang2, which is quite similar to Ang1, functions as an agonist/antagonist depending on the circumstances. Every member of the angiopoietin family, which includes Ang1 to Ang4, has a fibrinogen domain at the carboxyl end, accompanied by a coiled-coil “rope” and “super clustering” area at the amino end [18].

### 2.2. Angiopoietin-2 (Ang2)

Angiopoietin-2 (Ang2) is a growth factor that acts as an antagonist cytokine and belongs to the angiopoietin/Tie (tyrosine kinase with Ig and EGF homology domains) signaling pathway [19]. After the identification of angiopoietin-1 (Ang1), angiopoietin-2 was discovered through cDNA screening. It is a potent angiogenic factor required for in vivo angiogenesis and has different properties from the vascular endothelial growth factor (VEGF) [20]. Approximately 60% of the amino acids in Ang1’s 496-amino acid protein are also found in Ang2, but Ang2 possesses only eight of the nine mature cysteines present in Ang1. Ang2 has a secretion signal peptide, a coiled-coil domain at the NH2 end, and a fibrinogen-like domain at the COOH end. Ang2 operates in a more authoritarian and autocratic manner and exhibits tightly controlled expression compared to Ang1 [21]. Ang2 functions similarly to Ang1, except that it does not bind to Tie1; it binds to the Tie2 receptor with the same affinity and initiates its antagonistic effect. Inflammatory mediators, such as thrombin, as well as conditions like hypoxia and malignancy, will induce Ang2 expression [22].

In relation to other angiopoietins, previous studies have explained the notable structural differences between angiopoietin-3 and angiopoietin-4 compared to the mouse and human counterparts of angiopoietin-1 and angiopoietin-2. Research on the chromosomal localisation of all the angiopoietins in humans and mice has demonstrated that Ang3 and Ang4 seem to correspond to the respective gene loci in humans and mice. The structural difference of angiopoietin-3 and angiopoietin-4 suggests that their functions are distinct. Angiopoietin-3 functions as an antagonist, while angiopoietin-4 also acts as an agonist [23]. Nishimura et al. (1999) investigated Ang3 mRNA expression in lung cells and HUVECs. The results of the study concluded that there was a slight decrease in mRNA expression of Ang3 in HUVECs with vascular endothelial growth factor treatment [24]. Similarly, the role of Ang4 in tumor angiogenesis initiation and in activating Erk1/2 kinase in human glioblastoma multiforme cells was investigated [25]. 

### 2.3. Receptor Tyrosine Kinase 2 (Tie2)

Tie2 was identified as an endothelial growth receptor involved in the signal transduction of angiogenesis [26]. Ang1 interacts with Tie2, a receptor tyrosine kinase, through specific connections. Following the discovery that Tie2’s coreceptor, the related orphan receptor tyrosine kinase Tie1, functions as a coreceptor for Tie2, a novel molecular theory was proposed to explain the various signaling properties of Ang ligands. In fact, Tie2 signaling is inhibited by a complex formed by Tie1 and Tie2 on the surface of endothelial cells [27]. The Tie1/Tie2 interactions are primarily electrostatic in nature; however, the Ang ligands can alter them. Rapid phosphorylation, activation, Tie1/Tie2 dissociation, and Tie2 clustering all occur in the presence of the Tie2 agonist Ang1 [28]. Alternatively, Tie2 activation cannot be promoted by the receptor antagonist Ang2, as it does not affect the inhibitory Tie1/Tie2 complex. The Ang1-binding region of Tie2 is located within the initial immunoglobulin domain and the regions homologous to the epidermal growth factor within the 360-amino acid region of the receptor ectodomain [29].

### 2.4. Protein Kinase B (Akt)

Akt, also known as Protein Kinase B (PKB), regulates a number of essential cellular functions, including metabolism, apoptosis, migration, proliferation, and differentiation [30]. Akt influences a variety of pro- and anti-angiogenic factors. The Akt isoforms (Akt1, Akt2, and Akt3) have been suggested as potential therapeutic targets for diseases linked to angiogenesis, such as ischaemic injury and cancer [31]. 

Studies utilizing gene-knockout mice have shown that deletion of the Akt1 gene results in reduced organ size, impaired extra-embryonic vascular patterning, increased levels of apoptosis in certain cell types, impaired growth and development of the organism, placental hypotrophy, and a partially penetrant phenotype associated with higher fetal mortality [32]. Disruption of the Akt2 gene leads to insulin resistance and diabetes by compromising insulin signaling in skeletal muscle and the liver, resulting in mild growth deficits. Moreover, Akt3 knockout mice exhibit a primarily neurological phenotype and a smaller brain [33]. The significant functional overlap among Akt isoforms is highlighted by the severe growth retardation and other developmental problems that result in fetal mortality when both the Akt1 and Akt2 genes are deleted [34].

### 2.5. Phosphatidylinositol 3-Kinase (PI3K)

Phosphatidylinositol 3-kinases (PI3Ks) are a family of lipid kinases that catalyze the phosphorylation of phosphatidylinositides at their 3-hydroxyl position. They are involved in many cellular processes, such as survival, proliferation, differentiation, and nutrient absorption [35]. Despite having different substrate choices and regulation methods, at least eight members of the PI3K family have sequence homology in their kinase domains [36]. The four class I PI3Ks (α, β, γ, and δ isoforms) are the most well-characterized members of this family. They associate PI3K activity with a range of cell-surface receptors, including growth factor receptors and G protein-coupled receptors (GPCRs) [37]. There is strong evidence that many human malignancies have dysregulated PI3K/Akt signaling. For instance, the PIK3CA gene, which codes for the p110α subunit, is mutated in a broad range of malignancies, such as breast, colorectal, glioma, and gastric cancers, and is amplified and overexpressed in multiple cell lines, including cervical, gastric, and ovarian cancer cell lines [38]. The lipid phosphatase PTEN is the protein that is mutated in human cancer. It inhibits PI3K signaling adversely and adds to the increasing amount of evidence connecting the PI3K pathway to cancer. Thus, the blocking of PI3K, mainly the p110α subunit, represents a promising avenue for cancer therapy [39].

## 3. Role of Ang1-Tie2/AKT-PI3K in Angiogenesis

**Figure 2:**
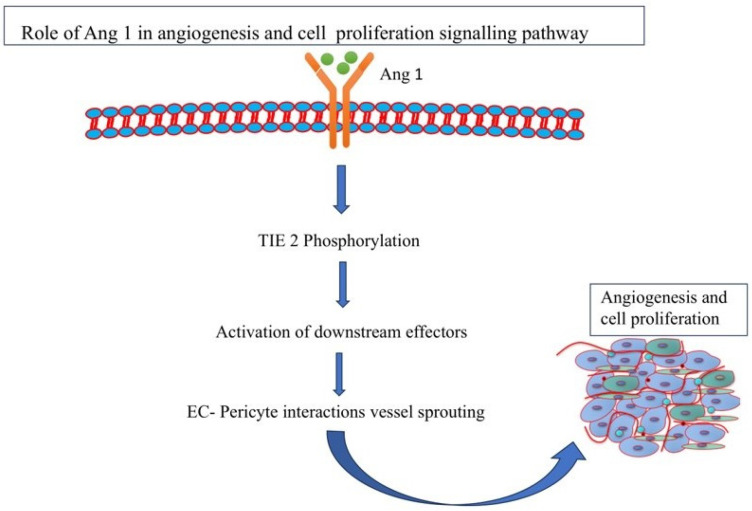
Role of Ang1 in the angiogenesis and cell proliferation pathway.

Angiogenesis is the process by which new blood vessels protrude from pre-existing vascular tubes or by which pre-existing blood vessels split apart due to intussusception (IA). It also refers to the formation of arteries and veins from pre-existing blood vessels. Three angiopoietins in humans (Ang1, Ang2, and Ang4) and one in mice (Ang3), are collectively known as the angiopoietins. Initially, it was discovered that Ang2 was an antagonist for Ang1 because it prevented Ang1 from activating Tie2 [40]. Ang1 activates the signaling pathway through the Tie2 receptor and stimulates vessel remodeling, cell proliferation, and angiogenesis (Figure 2). Previous studies revealed that, similar to Ang1-null animals, transgenic mice overexpressing Ang2 exhibited disturbed connections between endothelial cells (ECs) and peri-ECs. It also significantly interfered with the development of blood vessels in mouse embryos. Ang2 is only expressed at the sites of vascular remodeling in mature mice and humans [41]. Additionally, other angiogenic factors including nitric oxide and angiopoietins, are also modulated by the PI3K/Akt pathway. Research on PI3K/Akt networks has resulted in the development of inhibitors that are crucial for increasing the survival rate of cancer patients [42]. 

According to a study by Kim et al. (2000), Ang1 regulates endothelial cell survival via the PI3K/Akt signaling pathway. Human umbilical vein endothelial cells treated with the Tie2 receptor, which blocks Ang1-induced anti-apoptotic effects, showed an increase in PI3-kinase activity [43]. Similarly, it was found that the increase in endothelial nitric oxide synthase (eNOS) and the release of nitric oxide (NO) are required for Ang1-induced PI3K/Akt signaling. Treatment with recombinant Ang1 resulted in PI3-kinase-dependent Akt phosphorylation [44]. On the other hand, Ang2 functions as a suppressor, hastening vascular shrinkage in the absence of VEGF-A [45]. Ang2 upregulation is correlated with the aggressiveness of many human malignancies. Ang2 overexpression enhances the growth and angiogenesis of tumors in mice and many human cancerous cell lines. However, additional research has demonstrated that targeted Ang2 activation prevents the growth and metastasis of gliomas, mammary carcinomas, and lung carcinomas [46]. It was observed that Ang2 inhibits angiogenesis and prevents ECs from forming tubes in vitro in a chorioallantoic membrane (CAM) system [47].

## 4. Tie2-Mediated Signaling

**Figure 3:**
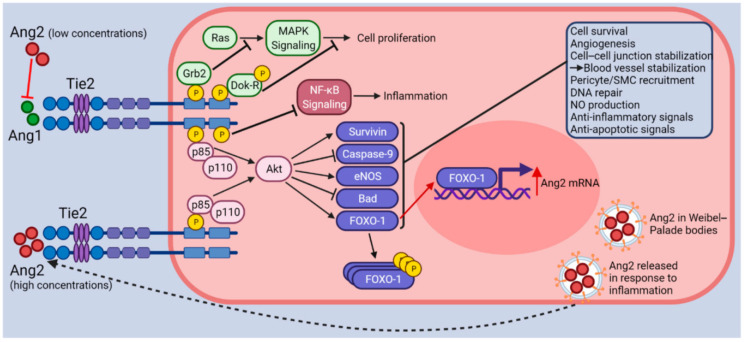
Ang-Tie2 Signaling pathway [53].

Tie2, which is predominantly expressed in endothelial cells, is the homologous receptor for Ang1 and Ang2, which bind to Tie2. The Tie2 receptor forms a dimer upon Ang binding and is phosphorylated at its C-terminal tyrosine residues [48]. Through the phosphatidylinositol 3-kinase (PI3K)-dependent pathway the Tie2 activates Akt. Therefore, it increases cell survival, reducing the release of the mitochondrial-derived activator of caspase (Smac), and suppressing the actions of caspases 3, 7, and 9 [49]. The Tie2 receptor binds to Ang1 and is constitutively activated by phosphorylation (p-Tie2) in the quiescent vasculature (Figure 3). This leads to the simultaneous deactivation of the forkhead transcription factor FOXO1 (also referred to as FKHR1) and stimulation of the downstream phosphoinositide 3-kinase (PI3K)/AKT signaling pathway. Thus, Ang1-mediated inhibition of FOXO1 via nuclear exclusion increases the expression of genes linked to vascular stability while suppressing factors like Ang2 that lead to vascular destabilization [50,51]. 

It was discovered that Ang2 inhibited the expression of VEGF-A in human glioma cell lines, a non-EC that was shown to have Tie2, but not in cancer cells that were Tie2-negative. On the transcriptional level, Ang2 altered the expression of VEGF, although hypoxia-inducible factor (HIF)-1 expression and HIF-DNA binding activity were decreased. Since Tie2 is essential to this regulatory loop, the Ang2-mediated down-modulation of VEGF was inhibited by small interfering RNA (siRNA)-mediated silencing of Tie2 [52].

## 5. Role of Flavonoids

Recently, the potential anti-angiogenic effects of numerous bioactive plant components have been investigated. Flavonoids, which are widely distributed in fruits and vegetables, are among the substances that have been most extensively examined. Flavonoids prevent angiogenesis and metastasis through regulation of several signaling pathways as shown in Table 1. Flavonoids possess potent anti-angiogenic actions by inhibiting the PI3-K/Akt, ERK1/2, NF-κB, and matrix metalloproteinase (MMP) signaling pathways, and by regulating the expression of VEGF and EGFR. There are many subclasses of flavonoids, including flavones, chalcones, isoflavones, and flavonols [54]. Numerous investigations have shown how closely the regulation of malignant growth cells, endothelial cells, and angiogenic factors is linked to flavonoids and related compounds [55,56,57]. 

The effect of naringenin on tumor growth and angiogenesis in human malignant melanoma was studied by Choi et al. in 2020. Naringenin treatment was administered to B16F10 and SK-MEL-28 cells. The results showed phosphorylation of ERK1/2 and inhibition of JNK MAPK. Whereas the in vivo experiments demonstrated a suppression of epithelial cells, as well as the sprouting and tube formation of microvessels. Furthermore, the RT-PCR analysis of treated cell mRNA showed an inhibition of ANG-2 expression [58]. The endogenous thrombospondins TSP-1 and TSP-2 are effective regulators of angiogenesis. Hypoxia-inducible factors (HIFs) and NF-κB play an important role in cancer cell proliferation and metastasis by promoting angiogenesis [59]. 

According to Shimazaki et al. in 2021, the role of phytocompounds on molecular mechanisms of angiogenesis-promoting and -suppressing factors were studied. In this study, HUVEC cells and human rAng1-producing 107-35 CHO cells or mouse DFAT-D1 cells were cultured. The flavonoids resveratrol, luteolin, and quercetin were administered in a dose-dependent manner, resulting in the suppression of COX-1, α-SMA, and Flk1 [60].

On the other hand, a comparable in vivo and in vitro investigation was conducted by Hana and Bawi (2022) to explore the possibility that hesperidin (Hsp) inhibited laryngeal cancer. Hsp prevented human Hep2 laryngeal cancer cells from metastasizing to the liver and lungs in an in vivo animal model. At relatively low concentrations (10 μM), Hsp significantly increased the apoptosis indicator annexin-V and reduced the angiogenic promoter angiopoietin-1 production in Hep2 cell culture. This investigation indicates that further research on Hsp could be a potential therapeutic approach for laryngeal cancer [61].

**Table 1:** Role of flavonoids in inhibiting different pathways of angiogenesis.

S.No	Phytochemical	Type of Cancer	Cell Line Used	Mechanism	Reference
1.	Kaempferol	Ovarian cancer	OVCAR-3 and A2780/CP70	↓HIF-1α, ↓AKT phosphorylation, ↓ ESRRA	[62]
2.	Myricetin	Breast cancer	MDA-MB-231 and 4T1	↓VEGFR2 and p38MAPK	[63]
3.	Luteolin	Gastric cancer	MGC-803, Hs-746T	↓Notch1/VEGF	[64]
4.	Epigallocatechin-3-gallate	Liver cancer	SMMC-7721 and HepG2	↓HIF-1α and VEGF	[65]
5.	Herbacetin	Malignant melanoma	A375 and Hs294T	↓EGFR, ↓AKT and ERK, ↓MMP9	[66]
6.	Fisetin	Breast cancer	4T1, MCF-7 and MDA-MB-231	↓Akt, P70, and mTOR, p-PI3K	[67]
7.	Galangin	Ovarian cancer	OVCAR-3 and A2780/CP70	↓HIF-1α, inhibited phosphorylation of Akt and ↓p70S6K	[68]
8.	Quercetin	Breast cancer	TAMR-MCF-7 cells	↓HIF-1α and AP-1, ↓VEGF secretion and Pin1	[69]
9.	Rhamnazin	Breast cancer	MDA-MB-231	↓VEGF-induced VEGFR2	[70]
10.	Delphinidin	Lung cancer	A549	↓CoCl2, ↓HIF-1α, ↓VEGF	[71]

## 6. Inhibition of PI3K/AKT Signaling Pathway by Different Flavonoids

The PI3K/AKT pathway is essential for cell growth, proliferation, and survival. Although this cellular signaling system is strictly controlled, excessive activity within it is frequently linked to numerous types of human malignancies and resistance to anti-cancer drugs [72]. Phosphatidylinositol 3-kinase (PI3K) is one of the key actors in these pathways and upstream targets. The membrane-bound phosphatidylinositol-(4,5)-bisphosphate (PIP2) is phosphorylated by the class I PI3Ks and transformed into phosphatidylinositol-(3,4,5)-trisphosphate (PIP3). PIP3 regulates growth, proliferation, and survival signaling by enlisting and activating the downstream kinase AKT, which contains the pleckstrin homology (PH) domain [73]. Numerous studies have shown that the flavonoids found in nature possess anti-cancer potential by inhibiting the PI3K/AKT pathways in cancer cells [74,75,76].

In addition, Rehan et al. (2020) studied the molecular docking of the ATP-binding site of PI3K, where flavonoids were screened virtually. The top 10 scoring flavonoids were chosen for posture analysis and assessment of binding strength. The results showed that all 10 selected flavonoids had the potential to function as PI3K kinase inhibitors and anti-cancer drugs [77]. On the other hand, in vitro research on the dichloromethane extract of *Ericameria nauseosa* revealed that it was responsible for reducing AKT activity in MM121224 human melanoma cells [78]. 

Li et al. (2010) found that the transcription factor forkhead box O3 (FOXO3a) is a downstream target of the PI3K/AKT pathway [79]. Breast cancer patients have a poor prognosis when FOXO3a is expressed at high levels in the cytoplasm, which is associated with Akt phosphorylation [80]. For instance, Lin et al. (2015) examined the anti-tumor effects of flavone, apigenin, and luteolin in breast cancer cells. The investigation showed that these polyphenols inhibited phosphoinositide 3-kinase (PI3K) and protein kinase B (PKB)/Akt and increased the expression of FOXO3a [81]. In contrast, Wang et al. (2021) studied the anti-tumor effects of astragalin, a natural flavonoid molecule, in stomach cancer cell lines using a xenograft mouse model. The results showed that astragalin strongly blocked the PI3K/AKT signaling pathway, increased the expression of apoptotic signaling proteins, and reduced cancer cell motility and invasion [82].

## 7. Safety of Flavonoids

Since many foods naturally contain a high amount of polyphenols and also have a long history of use in the diet. The flavonoids obtained from food are widely recognized as being safe for humans and will be consumed more if dietary recommendations for improving health include eating more plant-based foods [83]. However, the increasing use of foods and beverages naturally high in flavonoids, as well as foods enriched with these phytonutrients and dietary supplements containing them, has been promoted by research into the purported health advantages of flavonoids [84]. For instance, quercetin has been marketed as a dietary supplement, yet the daily consumption of this flavonol from food is only thought to be between 10 and 100 mg. Consumers may believe that flavonoid supplements are harmless to use since they are “natural”, and there is no evidence of quercetin toxicity from supplemental ingestion [85]. It is significant to highlight that older persons are more likely to utilize prescription drugs and dietary supplements than their younger counterparts. Therefore, research into the safety of consuming flavonoids in high doses is necessary, particularly in older individuals [86].

## 8. Conclusions

Evidence suggests that VEGF, Ang1, and Ang2 collaborate to modulate tumor angiogenesis through Tie2-dependent pathways. The regulation of angiogenesis, vascular remodeling, and vascular permeability by the Ang-Tie axis is crucial for maintaining vascular inactivity. Currently, the development of cancer angiogenesis therapy critically depends on the discovery of safe, affordable compounds based on or derived from the structure of flavonoids. Numerous flavonoids have undergone pharmacological analyses in vitro and in vivo, demonstrating a wide range of cellular and molecular processes mediating anti-angiogenic effects. Existing anti-angiogenic drugs used to treat cancer fall short of expectations in terms of safety and efficacy. Furthermore, natural plant compounds, such as flavonoids, can be utilized to treat cancer in combination with chemotherapy medications to regulate the proliferation, survival, and maintenance of cancer cells. Therefore, additional research is needed in the development of sustainable anti-angiogenic drugs. Development of novel nano-formulations could play an important role in the sustained release of flavonoids. Furthermore, novel derivatives of flavonoids can be synthesized to enhance the bioavailability and bioactivity efficacy. The development of novel, safer, and more effective treatments that inhibit the formation of new blood vessels and, as a result, cut off the supply of nutrients and oxygen to the tumor should be the goal of ongoing research in this field.

## Data Availability

Not applicable.
